# Assessment of Standards and Codes Dedicated to CFRP Confinement of RC Columns

**DOI:** 10.3390/ma12152390

**Published:** 2019-07-26

**Authors:** Stefan Kaeseberg, Dennis Messerer, Klaus Holschemacher

**Affiliations:** Leipzig University of Applied Sciences, Structural Concrete Institute (IfB), Karl-Liebknecht-Str. 143, 04277 Leipzig, Germany

**Keywords:** reinforced concrete, columns, confinement, CFRP, load bearing capacity, standards

## Abstract

Reinforced concrete (RC) columns are often placed under confinement to increase their strength and ductility. Carbon fiber reinforced polymer (CFRP) materials have recently been recognized as favorable confinement systems. At present, a number of national standards and codes dedicated to the design of concrete components strengthened with CFRP in general and CFRP confinement in particular are available. These sets of rules provide design equations for confined reinforced concrete columns with circular and rectangular cross sections. Most of the standards and codes exhibit significant differences, including the used predictive models, limitations, observed effects and covered loading conditions. In this paper, five international standards and design guidelines are introduced and discussed. The purpose is to present a constructive and critical assessment of the state-of-the-art design methodologies available for CFRP confined RC columns and to discuss effects not previously considered properly. Therefore, some recent research efforts and findings from the Leipzig University of Applied Sciences are also introduced. The obtained data is used for a comparative study of the guideline predictive equations. Furthermore, it is shown that some new findings concerning the rupture strength and the maximum strength plus accompanying axial strain of a CFRP confined column are suitable to improve the current guidelines.

## 1. Introduction

Confinement is generally applied to concrete members in compression to increase their strength and ductility. In addition to conventional transverse tie reinforcing steel, fiber reinforced polymer (FRP) materials have recently been recognized as favorable confinement devices. FRP consists of strengthening fibers (e.g., carbon fibers) in a resin matrix. FRP confinement can be applied with a fiber orientation transverse to the longitudinal axis of the concrete member. Due to FRP confinement, the concrete’s lateral expansion can be efficiently restricted in cases of compressive axial deformations. Therefore, the elastic FRP resistance response generates a steadily increasing lateral compressive stress state of the concrete, leading to a structural upgrade of the member’s core and providing sufficient deformability.

So far, extensive work in both the experimental and analytical fields has been conducted, and various experimental research studies have been carried out to understand the increase in strength and strain when using FRP jackets, e.g. [1,2,3,4,5,6,7,8,9,10,11,12,13,14]. In general, models provide equations for the calculation of the new concrete strength *f*_cc_ and accompanying axial strain *ε*_ccu_. Typical forms are:

(1) $$f_{\mathrm{cc}}{\;=\;f}_{c}{\;+\;k}_{1}\;\cdot {\;f}_{\mathrm{lj}}$$

(2) $$\varepsilon_{\mathrm{ccu}}{\;=\;\varepsilon }_{c0}\;\cdot {\;k}_{2}{\;+\;\varepsilon }_{c0}\;\cdot {\;k}_{3}\;\cdot \;\frac{f_{\mathrm{lj}}}{f_{c}}\;\cdot \;{\left(\frac{\varepsilon_{\mathrm{ju}}}{\varepsilon_{c0}}\right)}^{k_{4}}$$

where *f*_c_ is the mean value of the unconfined concrete strength, *ε*_c0_ is the peak strain of the unconfined concrete, *f*_lj_ is the confinement pressure provided by the FRP jacket, *ε*_ju_ is the rupture strain of the FRP jacket at the column, and *k*_1_–*k*_4_ are factors swaying the impact of *f*_lj_ on *f*_cc_ and *ε*_ccu_.

Equations (1) and (2) highlight the importance of *f*_lj_, which for a column with a circular cross section is determined by:

(3) $$f_{\mathrm{lj}}\;=\;\frac{2\;\cdot {\;t}_{j}\;\cdot {\;E}_{j}\;\cdot {\;\varepsilon }_{\mathrm{ju}}}{D}$$

where *E*_j_ is the modulus of the composite material, *t*_j_ is the FRP thickness, and *D* is the diameter of the circular cross section.

**Figure 1:**
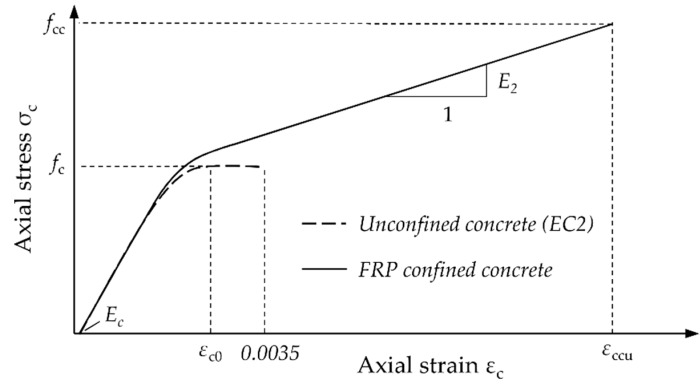
Stress-strain model for FRP confined concrete according to Lam and Teng [15].

Equations (1) and (2) are used to characterize the behavior of a column under concentric compression, or when the eccentricity present in the column is very small. Furthermore, proper confinement can provide significant strength enhancement for members subjected to combined compression and flexure as well. For this case, a model is necessary to describe the entire material behavior of confined concrete under compressive stress. In general, a stress-strain (*σ*_c_-*ε*_c_) curve consisting of a parabolic first portion and a straight-line second portion (second modulus) is introduced. An example is given by the stress-strain model of Lam and Teng [15]:

(4) $$\sigma_{c}\;=\;\{\begin{array}{c} E_{c}\;\cdot {\;\varepsilon }_{c0}\;-\;\frac{{\left(E_{c}\;-{\;E}_{2}\right)}^{2}}{4\;\cdot {\;f}_{c}}\;\cdot {{\;\varepsilon }_{c0}}^{2}\;\left(0\;\leq {\;\varepsilon }_{c0}\;\leq {\;\varepsilon }_{t}\right)\\ f_{c}{+\;E}_{2}\;\cdot {\;\varepsilon }_{c0}\;\left(\varepsilon_{t}\;\leq {\;\varepsilon }_{c0}\;\leq {\;\varepsilon }_{\mathrm{ccu}}\right) \end{array}$$

where *E*_2_ is the second modulus, *E*_c_ is the modulus of elasticity, and *ε*_t_ is the transition between the parabolic curve and the straight-line second portion. Lam and Teng’s stress-strain model is illustrated in Figure 1.

Usually, a stress block factor *α*_1_ is introduced to simplify the design procedure. For instance, Jiang [16,17] proposed the following equation:

(5) $$\alpha_{1}\;=\;1.17\;-\;0.2\;\cdot \;\frac{f_{\mathrm{cc}}}{f_{c}}$$

**Figure 2:**
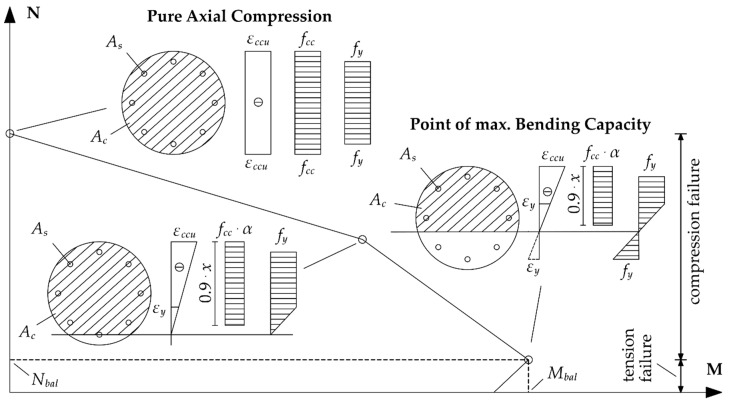
Design procedure for a fiber reinforced polymer (FRP) confined reinforced concrete (RC) column subjected to combined compression and bending.

Taking into consideration the assumptions above, moment-normal force (M-N) diagrams can be introduced by satisfying the force equilibrium and strain compatibility, utilizing Equations (4) or (5). An example of an entire design procedure is presented in Figure 2.

Corresponding to Mandate M/515 EN of the European Commission [18], the second generation of the Eurocode 2 [19] will prospectively include the strengthening of existing structures with FRP. To date, several countries and institutions have introduced national standards, codes, and guidelines to provide frameworks for the design of the FRP confinement of reinforced concrete (RC) columns, commonly for strengthening purposes. Up to now, there is no proper and up-to-date review of those, taking into consideration the differing fundamental scientific models and assumptions. In this paper, current examples, representing the international state of the art, are introduced and discussed. The aim is to present a constructive and critical assessment of the design methods used including the suggested boundaries and limitations. In addition, as yet unconsidered or insufficiently considered effects are also discussed. It should be noted that this paper is limited to the application of carbon fiber reinforced polymers (CFRP) on columns with circular cross sections.

## 2. Overview of Contemporary Standards and Guidelines

### 2.1. General Information

Table 1 shows the current standards and guidelines considered in this paper. Besides recommendations concerning confinement, the provisions enable the use and design of different FRP strengthening materials and methods, such as CFRP-plates, near surface mounted CFRPs, and FRP sheets for diverse purposes such as flexural or shear strengthening. In some cases, applications are not limited to RC structures, but also provide recommendations for the retrofitting of masonry and steel components. Such advice is, for instance, available in S806-12 [20] or CNR-DT 200 R1/2013 [21]. The considerably varying number of pages, seen in Table 1, reveals a first indication concerning the significance of confinement in the various standards. While the confinement section in the Canadian standard S806-12 [20] hardly fills one page, more detailed provisions are presented in the Chinese code [22] and German guideline [23].

Similar findings are evident in Table 2, where general information on the codes is described, including their limitations and the used models. While the German guideline defines boundaries based on the mean value of the unconfined concrete strength (*f*_c_), maximum eccentricity (*e*_0_/*D*), and maximum column slenderness (*λ*), such limitations are absent in other codes. However, all codes, except for the DAfStb guideline, enable the design of columns with square and rectangular cross sections.

It is noteworthy that only ACI 440.2R-17 and the German guideline allow for a serviceability limit state (SLS) design, despite the fact that constant compression stress is strongly increased in a confined RC column, which significantly enhances additional effects, such as concrete creep.

The Italian code enables discontinuous wrapping over the column’s height, which is prohibited or not mentioned in other standards. Finally, ACI 440.2R-17 and CNR-DT 200 R1/2013 enable the design of the confined column’s resistance under pure axial compression, while all other standards focus solely on the more common case of combined axial compression and bending. Curiously, in Italy the superordinate code for the design of RC structures, EN 1992-1-1 [19], requires the consideration of a minimum value for force eccentricity *e*_0_ of 20 mm for non-slender columns as well. Hence, design for pure axial compression is impossible.

### 2.2. Strength Reduction and Material Safety Factors for the Different Guidelines

While all guidelines have a consistent approach to the load amplification factors, strength reduction factors are considered in two different ways. The American Concrete Institute (ACI) uses strength reduction factors φ, which multiply the computed overall nominal capacity. In all other standards and guidelines, strength reduction factors or material safety factors *γ* are applied individually to each of the material components of the members during the calculation of the resistance.

Table 3 shows the strength reduction factors and partial safety factors for FRP materials used by the various guidelines. Furthermore, input values of material properties and additional safety factors from important related standards (e.g., EN 1992-1-1 [19] in case of the Italian code and the German guideline), such as the partial safety factors for concrete and steel, are also introduced.

**Figure 3:**
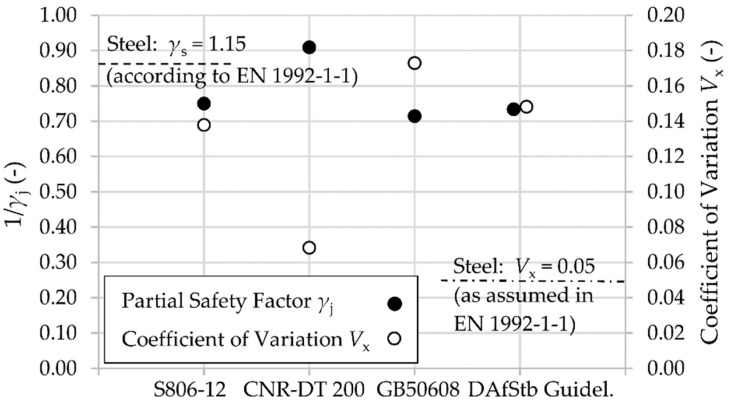
The partial safety factor *γ*_j_ introduced in the designated standard or guideline and the corresponding coefficient of variation *V*_x_.

Figure 3 shows the values of the partial safety factor associated with the FRP jacket, *γ*_j_, in the various codes. Furthermore, the presumed coefficient of variation *V*_x_ of the rupture strain *ε*_FRP_ for the CFRP is also displayed. The coefficient of variation is derived, via iteration, from Equation (6) in accordance with *fib* (International Federation for Structural Concrete) bulletin 80 [27]:

(6) $$\gamma_{j}\;=\;\frac{\exp(-1.645\;\cdot {\;V}_{x})}{\exp(-\alpha_{R}\;\cdot \;\beta \;\cdot {\;V}_{x})}\;\cdot {\;\gamma }_{\mathrm{Rd}1}\;\cdot {\;\gamma }_{\mathrm{Rd}2}$$

where *α*_R_ is the sensitivity factor (*α*_R_ = 0.8), *β* is the reliability factor (*β* = 3.8), *γ*_Rd1_ is a factor accounting for model uncertainties, and *γ*_Rd2_ is a factor accounting for geometrical uncertainties.

For the case of circular CFRP confinement, *γ*_Rd1_ is assumed to be 1.1 and *γ*_Rd2_ is predicted to be 1.0 because of the fact that geometrical uncertainty is limited to the diameter *D* and minor changes of *D* can occur without significant discrepancies. Figure 3 shows that reduction due to safety factors varies between 10 and 30%. In addition, the related coefficient of variation *V*_x_ probably presumed in codes is noteworthy as well. While the Italian code employs a comparatively low variation of FRP strength, which is comparable to steel, the Chinese GB 50608 assumes a considerably higher *V*_x_. All partial safety factors seem to originate from common axial flat coupon tensile tests. The fact that the factors are also applicable for bending or shear design supports this assumption. This approach is questionable, taking into consideration the significantly different stresses appearing under bending and shear in FRP sheets compared to confinement applications (e.g., Section 2.3). Furthermore, all codes and standards introduce additional factors and partial safety factors depending on the type of composite material, manufacturing process, method of application, and environmental conditions. Table 3 provides an overview.

### 2.3. Rupture Strain of a CFRP Jacket and Confinement Pressure Provided by an FRP Jacket

As indicated by Equation (3), in addition to the column diameter *D* and the FRP jacket thickness *t*_j_, the rupture strain of the CFRP jacket under confinement *ε*_ju_ has a very strong impact on the confinement pressure *f*_lj_. According to the current state of the art, *ε*_ju_ should be taken as the actual hoop rupture strain measured in the FRP jacket rather than the FRP materials’ ultimate tensile strain *ε*_FRP_. The reason for this is that at the rupture of an FRP jacket under confinement, the hoop strain obtained in the jacket *ε*_ju_ is generally considerably smaller than the ultimate tensile strain found from flat coupon tensile tests *ε*_FRP_. On this basis, Lam and Teng [15] established an FRP efficiency factor *k*_ε_, defined by:

(7) $$\varepsilon_{\mathrm{ju}}{\;=\;\varepsilon }_{\mathrm{FRP}}\;\cdot {\;k}_{\varepsilon }$$

In Table 4, different values for *k*_ε_, proposed in codes and guidelines, are specified. Remarkably, two in five standards still consider *k*_ε_ = 1.0, despite scientific recommendations [28,29]. On the other hand, in ACI 440, *k*_ε_ is recommended as 0.55, and in the Chinese code (CFRP) as well as in the German guideline, *k*_ε_ is assumed to be 0.50. At this point, the DAfStb-Guideline seems to offer the most advanced approach, since the characteristic value *k*_εk_ is used for design. It is related to the characteristic 5-percentile value obtained from test results and, therefore, it can acknowledge the spreading (material property variation) of the rupture hoop strain for confinement applications. Furthermore, in order to realize a proper and coherent limit state method, a characteristic hoop rupture strain *ε*_juk_ is deployed.

In fact, the value introduced for *k*_εk_ = 0.25 seems very low. The reason for this is longitudinal steel reinforcement. Some research groups (e.g., Pellegrino and Modena [30]) proposed that longitudinal reinforcement has an impact on *k*_ε_ due to the fact that external FRP confinement in columns provides additional restraints for vertical steel bars. This leads to further strains concentrated inside the confinement, causing a further reduction of *k*_ε_ Niedermeier [26] followed this proposal and suggested a mean value *k*_ε_ = 0.50 and a characteristic value *k*_εk_ = 0.25. This procedure was adopted in the DAfStb-Guideline.

In addition to Equation (7), S806-12 and CNR-DT 200 introduced further limits (Table 4). The explanation for these limits was to avoid excessive cracking or loss of shear integrity. Unfortunately, the scientific basis of the limit values is not described further. The Chinese code and German guideline do not suggest any limits.

**Figure 4:**
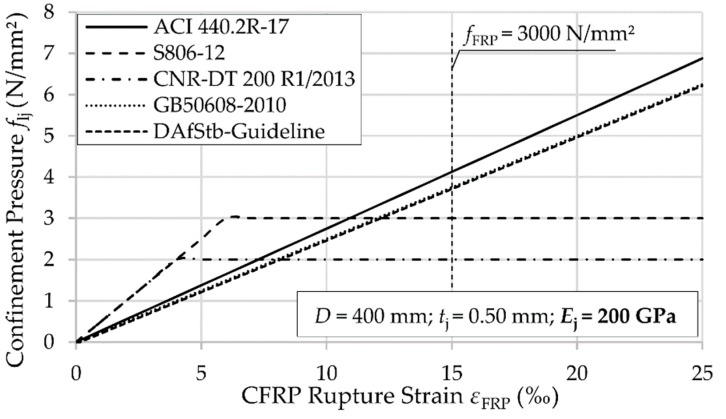
Confinement pressure *f*_lj_ depicted as a function of CFRP rupture strain *ε*_FRP_ (derived from flat coupon tests) when the modulus of the composite material is *E*_j_ = 200 GPa.

The implications of the different calculations of *ε*_ju_ on *f*_lj_ are shown in Figure 3 and Figure 4 for a circular cross section with *D* = 400 mm. For comparison purposes, *f*_lj_ was calculated without any strength reduction factor or partial safety factor. In Figure 4, a common elastic modulus *E*_j_ = 200 GPa of the composite material was chosen. For a typical maximum FRP strength, *f*_FRP_ = 3000 N/mm² at 15‰ rupture strain, the calculated *f*_lj_ varies between 2 and 4.2 N/mm². Using the Canadian or Italian codes, the particular complementary limits (Table 4) are decisive. Thus, the achievable *f*_lj_ remains, especially in case of Italian code, far below the pressure levels reached with ACI 440 or GB 50608.

**Figure 5:**
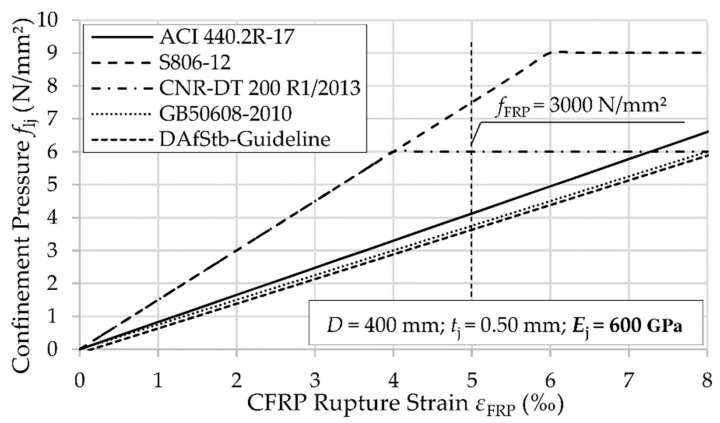
Confinement pressure *f*_lj_ depicted as a function of CFRP rupture strain *ε*_FRP_ (derived from flat coupon tests) when the modulus of the composite material is *E*_j_ = 600 GPa.

On the contrary, in Figure 5 where *E*_j_ = 600 GPA, the results are different. In this case, when *f*_FRP_ = 3000 N/mm², the confinement pressures vary between 3.5 and 7.5 N/mm². The *f*_lj_ presumed in accordance to S806-12 is twice as high as the *f*_lj_ calculated with the Chinese approach, with the complementary limit not being reached. This leads to the conclusion that in the case of the Italian or Canadian code, an FRP sheet with a high *E*_j_ should be chosen, so a high utilization of the FRP material is feasible. This fact is in total opposition to the approaches suggested in ACI 440, GB 50608, and the DAfStb-Guideline.

### 2.4. Maximum Confined Concrete Compressive Strength and Maximum Concrete Strain

#### 2.4.1. Compressive Strength

In Section 1 (Introduction), the common equation to predict the maximum confined concrete compressive strength *f*_cc_ was introduced (Equation (1)). It is obvious that *f*_cc_ strongly relies on the factor *k*_1_, so Table 5 gives an overview of the *k*_1_ introduced in the respective codes and guidelines. Table 5 confirms the fundamental differences among codes regarding the calculation of *k*_1_. ACI 440 and the German DAfStb-Guideline propose a constant factor. Such an approach is in accordance with previous studies [15,26]. Interestingly, in DAfStb-Guideline, the characteristic value of *k*_1_ is used for design. The other codes deploy the mean value.

In contrast, the Chinese standard introduces the ratio between the stiffness of the FRP jacket and the unconfined characteristic concrete strength *f*_ck_ via *β*_j_, to calculate *k*_1_. This procedure follows suggestions made by Teng et al. [25]. Incomprehensibly, *f*_ck_ is used for calculation. Especially for old and matured concrete, as expected in the case of strengthening, *f*_ck_ can be considerably smaller if compared to the mean value *f*_c_ due to higher variability of concrete strength during sampling. Consequently, this approach leads to uncertain results if compared to a procedure where *f*_c_ is utilized.

In turn, the Canadian code S806-12 identifies the dependence of *k*_1_ on *f*_lj_. This approach is similar to a proposal made by Samaan et al. [31]. In the Italian code, Equation (1) is altered, and *k*_1_ must be multiplied by the ratio of the confinement pressure to the unconfined concrete strength. Such a proceeding is similar to findings in Reference [32]. For the calculation of this ratio, the design value *f*_cd_ = *f*_ck_/*γ*_c_ is used instead of *f*_c_. Again, such a procedure can cause uncertain results if the coefficient of variation of the concrete’s compressive strength is significant.

Finally, Table 5 points out that in five standards and guidelines, four completely different approaches for the assumption of *k*_1_ can be found. Furthermore, it is worth mentioning that in the German DAfStb-Guideline, Equation (1) has been altered to implement the consideration of internal transverse reinforcement (ties or spirals) in the calculation of the confinement pressure. This approach is in accordance with proposals released by several researchers, e.g. [33,34,35]. No other standard considers transverse reinforcement.

#### 2.4.2. Concrete Strain

In addition to the increased concrete strength, confinement also leads to a highly increased maximum strain of the confined concrete member. In Table 6, a brief overview is given on how the various standards calculate *ε*_ccu_.

**Figure 6:**
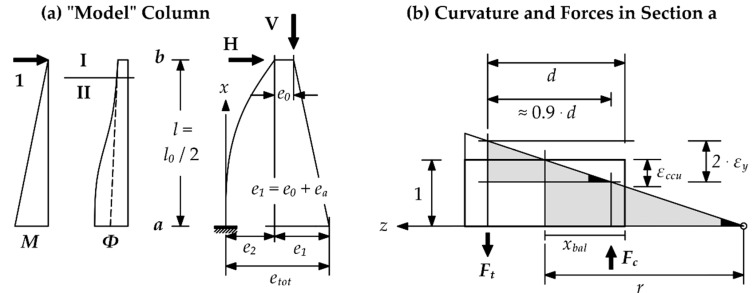
Effect of *ε*_ccu_ on the column behavior under combined axial compression and bending [36]. (**a**) Model representation of a column exposed to normal force and bending moment. (**b**) Curvature and forces in Section a.

Once more, significant differences between the codes (especially the Canadian approach) are recognizable. However, an accurate value of *ε*_ccu_ is mandatory to predict the behavior of a confined column under combined axial compression and bending. This issue is visualized in Figure 6, which describes the strain distribution of a confined RC column at the point of maximum bending capacity (balance point). It shows that *ε*_ccu_, which determines the height of the compression zone *x*_bal_ and the curvature *ϕ*_bal_ of the column, is important to determine the complementary force eccentricity *e*_2_, used to recognize second order effects.

### 2.5. Example Calculation

**Figure 7:**
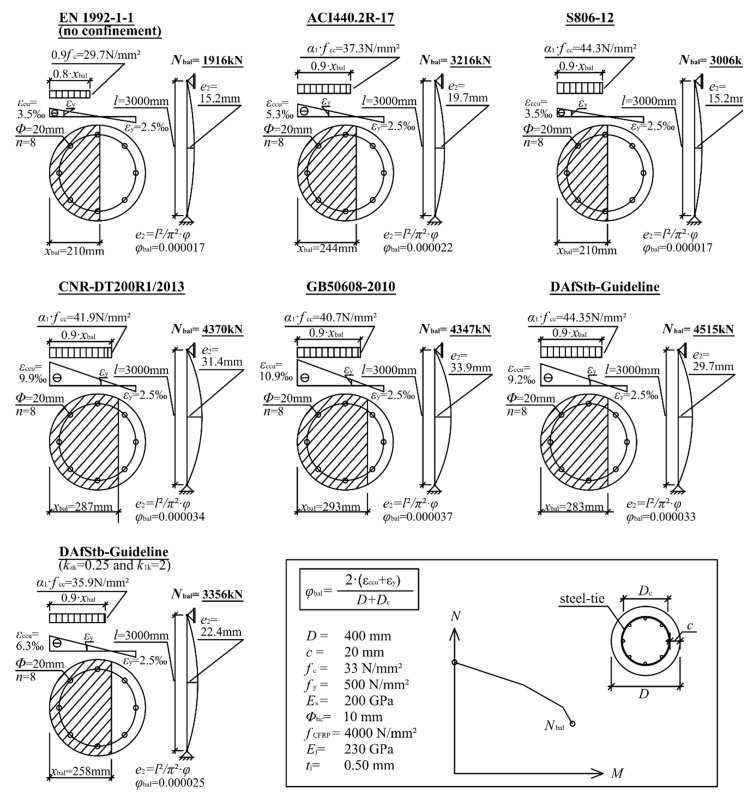
Stress-strain distribution of a CFRP confined RC column at the balance point, according to the considered codes and guidelines.

In Figure 7, a comparative overview for an example CFRP confined RC column is exhibited, assuming that the column is under combined axial compression and bending. The figure shows the strain distribution according to the particular code or standard, as well as the related stress distribution in the compression zone (stress block calculated according to Equation (5)) at its balance point. In addition, the respective axial load bearing capacity *N*_bal_ is mentioned. Finally, the related complementary force eccentricity *e*_2_ is displayed to explain the implication of the curvature *ϕ*_bal_ For better classification, the column was designed without CFRP confinement in accordance with EN 1992-1-1 [19]. All calculations were carried out by using mean values of the material properties. Strength reduction or material safety factors were not used. As a particularity, the German DAfStb-Guideline was calculated twice. In the first calculation, the mean values of *k*_1_ and *k*_ε_, in the second case, the characteristic values, *k*_1k_ and *k*_εk_, were used.

Figure 7 reveals a vast range of results calculated for the same basic column. While S806-12 predicts a moderate *N*_bal_ and a small *e*_2_, the DAfStb-Guideline (mean values) approach leads to an *N*_bal_ 2.35 times higher than determined for an unconfined column and predicts an *e*_2_ over twice as high as that calculated with S806-12. Then again, the German DAfStb-Guideline approach is the only one to stipulate the usage of characteristic values for *k*_1_ and *k*_ε_.

This fact reduces *N*_bal_ by about 25%. The biggest pressure zone is predicted by the Chinese code, covering 78% of the cross section. Due to a low *ε*_ccu_, the Canadian standard reveals the smallest compression zone, covering only 53% of the cross section.

### 2.6. Conclusion on Current Standards and Guidelines

Finally, it can be concluded that the considered codes and standards provide significantly different approaches to predict confinement effects. Almost all values and equations are dissimilar. This leads to the different and contrary results in design, as presented above.

Major contradictions and dissimilarities are located in:

− the definition of the partial safety factors;
− the commitment of the FRP efficiency factor *k*_ε_;
− the determination of *k*_1_; and
− the estimation of the maximum longitudinal concrete strain.

Furthermore, as mentioned earlier, in most cases the additional effects of reinforcing elements, like ties or spirals, cannot be considered. This can be related to the limited experimental data on the field of FRP confined real-size RC columns. These limits have not allowed for the appropriate implementation of key effects in the current models.

## 3. Experimental Findings

### 3.1. Current Research Emphasis

The codes and guidelines considered in this paper represent the state of research achieved in 2010. At this time, an extensive research program regarding CFRP confined short concrete columns has been launched at Leipzig University of Applied Sciences. The entire experimental program, further cited and used data from the literature, and particular results are described in References [37,38,39]. Concerning the discrepancies and differences discussed in the previous sections, the program revealed some remarkable findings regarding the crucial rupture strain *ε*_ju_, the entailed partial safety factor *γ*_j_, the prediction of *k*_1_, and the calculation of *f*_cc_ and *ε*_cc_ for confined plain and reinforced concrete columns.

### 3.2. Results Concerning FRP Rupture Strain of the Jacket and Accompanied Partial Safety Factors

**Figure 8:**
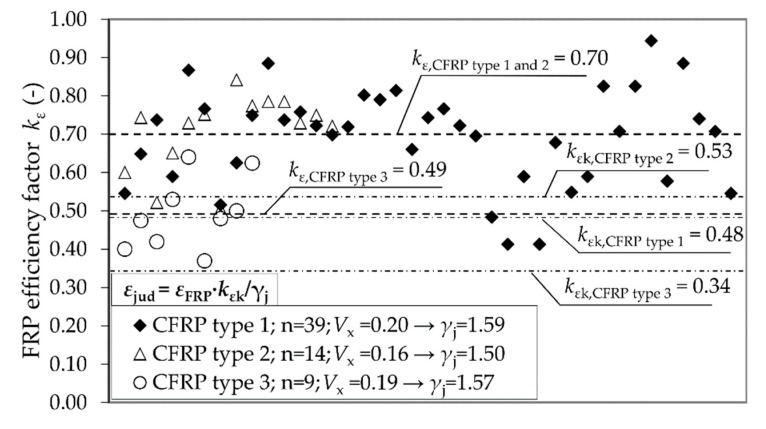
Values for *k*_ε_ determined from tests with different CFRP materials, calculated characteristic values *k*_εk_ (according to EN 1990), and partial safety factors *γ*_j_ (according to *fib* Bulletin 80 [27]).

Our own investigations [37] confirmed the proposal of Lam and Teng [15], who suggest working with a reduction factor *k*_ε_. In almost all cases, the rupture strain reached by the CFRP system was considerably lower than the ultimate tensile strain found from flat coupon tensile tests. Hence, a factor *k*_ε_ < 1.0 should be mandatory. While Lam and Teng—as well as ACI 440, GB 50608, and the DAfStb-Guideline—suggest a common, universally valid reduction factor for CFRP systems; our own tests show that there are significant differences between the carbon fibers used. The average value for three different CFRP systems differed remarkably between *k*_ε_ = 0.49 and *k*_ε_ = 0.70 (Figure 8). Despite a large spread of the test results, it was also possible to obtain characteristic values *k*_εk_ (in accordance with EN 1990). The test results acknowledged the DAfStb guidelines procedure to work with *k*_εk_. The use of a mean value *k*_ε_, as suggested in GB 50608, can therefore also be uncertain. In summary, efficiency factors should depend on the CFRP material and must be employed carefully.

Our own investigations with CFRP confined reinforced specimens did not confirm the assumption suggested in [26]; that longitudinal reinforcement has an additional, negative effect on *ε*_ju_. In tests, the longitudinal reinforcement remained without impact. It can be concluded that the reduction factor *k*_ε_ stays the same whether longitudinal reinforcement is deployed or not. This tendency was also observed in [40]. Especially in case of the German guideline, this finding leads us to the conclusion that a significant higher *k*_εk_ of approximately 0.50 (depending on the FRP material) can usually be used in Equation (7). The proposed value of *k*_εk_ = 0.25 is much too conservative for design matters and provokes an unnecessary loss of load bearing capacity, as seen in Figure 7.

Furthermore, this investigation succeeded in deriving the particular partial factors *γ*_j_ for the utilized CFRP materials. Equation (6) was used for the calculation. As explained in Figure 8, variation coefficients *V*_x_ vary remarkably between the CFRP-materials used. Hence, *γ*_j_ varies as well and should be determined separately for each FRP system, for instance within a technical approval procedure.

During the derivation of the displayed partial factors with Equation (6), *γ*_Rd1_ was predicted with a value of 1.20 due to the fact that model uncertainties are very high, but comparable with models for shear design. In contrast, *γ*_Rd2_ was determined with a value of 1.0. For columns with a circular cross section, geometrical uncertainties are small since *k*_ε_ persists at a constant value independent of the column diameter.

Finally, the calculated safety factors are much higher than those suggested by current codes and guidelines (Table 3). The reason for this, as has already been explained in Section 2.1, is that current partial safety factors originate from flat coupon tests only. This is a questionable and potentially unsafe procedure. *γ*_j_ depends on *V*_x_ of the FRP jacket’s hoop strain applied to the column perimeter. The same applies to the characteristic values of the FRP strength or FRP rupture strain. To accomplish a proper limit state design method, a value according to Equation (8), instead of a characteristic value derived from flat coupon tests, as used in the Chinese and Italian codes, should be employed:

(8) $$\varepsilon_{\mathrm{jud}}\;=\;\frac{\varepsilon_{\mathrm{juk}}}{\gamma_{j}}\;=\;\frac{\varepsilon_{\mathrm{FRP}}\;\cdot {\;k}_{\varepsilon k}}{\gamma_{j}}$$

### 3.3. Results for the Maximum Confined Concrete Compressive Strength and Maximum Concrete Strain

As is explained in Section 2.4, current codes and guidelines typically use a factor *k*_1_ to predict the influence of the confinement pressure on the confined concrete’s maximum compression strength *f*_cc_. In ACI 440 and DAfStb-Guideline a constant factor is used. Nevertheless, research efforts of, for example, Xiao and Wu [4] suggest that, in addition to *f*_lj_, the unconfined concrete cylinder strength *f*_c_ is of importance too.

Our own findings confirm these claims. From our work, it is obvious that if *f*_lj_ is deployed in relation to *f*_c_, comparably high consistency regressions can be found to explain *f*_cc_ and *ε*_ccu_. The following equations (Equations (9) and (10)) illustrate our proposal. It should be noted that these equations are designed for a limit state method (calculation of characteristic strength *f*_cck_), and that the procedure also enables the consideration of transverse reinforcement (via *f*_lk(j+w)_):

(9) $$f_{\mathrm{cck}}{\;=\;f}_{\mathrm{ck}}\;+\;30\;\cdot \;\ln\;\left(\frac{f_{\mathrm{lk}(j+w)}}{f_{c}}\right)\;+\;63\;\mathrm{if}\;0.75\;\geq \;\frac{f_{\mathrm{lk}(j+w)}}{f_{c}}\;\geq \;0.125$$

(10) $$\begin{array}{c} \varepsilon_{\mathrm{ccu}}{\;=\;\varepsilon }_{c0}\;\cdot \;1.75\;+\;0.05\;\cdot \;\frac{f_{\mathrm{lk}(j+w)}}{f_{c}}\\ \mathrm{with}\;f_{\mathrm{lk}(j+w)}{=\;f}_{\mathrm{ljk}}+\;\frac{1}{2}\;\cdot {\;\rho }_{w}\;\cdot {\;f}_{\mathrm{yk}}\;\cdot {\;k}_{e} \end{array}$$

where *f*_cck_ = is the characteristic value of the confined concrete strength, *f*_lk(j+w)_ is the characteristic confinement pressure provided by the FRP jacket and the transverse reinforcement, *ρ*_w_ is the ratio of transverse steel to the volume of concrete core, *f*_yk_ is the characteristic value of yield strength of transverse steel, and *k*_e_ is a factor to consider the different zones of influence of internal hoops and external FRP jacket (e.g. Table 5→DAfStb-Guideline).

Tests revealed the significant impact of transverse reinforcement. Spirals, in particular, provide a substantial confinement pressure, so the German guideline approach could be confirmed. In Equations (9) and (10), the ratio between confinement pressure and mean value of unconfined concrete strength is introduced, so the spread of *f*_c_, as frequently appears in old RC buildings, can be sufficiently considered. This approach can be regarded as safer and more accurate than proceedings that use values like *f*_ck_ or *f*_cd_ (e.g., GB 50608 or CNR-DT 200).

Moreover, the proposals described in the Chinese and Canadian code (Section 2.4.1) could not be confirmed by our tests. The sole dependency of *k*_1_ on the stiffness of FRP confinement (e.g., introduction of the factor *β*_j_ in GB 50608) or the sole reliance of *k*_1_ on *f*_lj_ (e.g., the S 806 approach) could be refuted.

## 4. Conclusions

Design approaches for FRP confinement of RC columns from five international design guidelines were presented, reviewed, and compared. Besides general information and limitations, the different procedures to predict partial safety factors, the confinement pressure *f*_lj_, and the design equations for the maximum axial compressive strength *f*_cc_ and ultimate axial strain *ε*_ccu_ of FRP confined RC members with circular cross-sectional shapes were outlined. These investigations revealed significant dissimilarities, leading to different and mostly contrary results among codes and guidelines. These differences may have developed historically, for instance, due to different intensity and prioritization of research in each country. This indicates the need for more intensive exchange, especially between the researchers involved in the standardization process and the determination of unified fundamentals and approaches. It has been shown that some new findings, obtained from elaborate tests carried out at Leipzig University of Applied Sciences, are suitable to improve current approaches. These include suggestions concerning a proper prediction (in accordance with the limit state method) of the crucial FRP hoop strain *ε*_ju_ and the entailed partial factor *γ*_j_. However, further research efforts are still necessary. Notably, loading conditions with combined axial compression and bending have not been sufficiently examined. Therefore, large scale tests with realistic longitudinal and transverse reinforcement, different concrete properties, varying FRP materials, and a proper test setup with sophisticated measurement methods are necessary to fully understand the complex interactions between all reinforcing parts and the strain distribution over the entire cross section during all relevant limit states.

## Figures and Tables

**Table 1:** Overview of reviewed standards, codes and guidelines.

Title	Country	Publishing Institution	Introduced	Confinement Section Length (pages)	Reference
ACI 440.2R-17	USA	American Concrete Institute (ACI)	2017	4	[24]
S806-12	Canada	Canadian Standards Association (CSA)	2012	1	[20]
CNR-DT 200 R1/2013	Italy	Advisory Committee on Technical Recommendations for Construction (CNR)	2014	6	[21]
GB 50608-2010	China	Standardization Administration of the People’s Republic of China	2011	8	[22]
DAfStb-Guideline	Germany	German Committee for Structural Concrete (DAfStb)	2012	5	[23]

**Table 2:** General information for the various standards, including limitations and the models used.

Standard/Guideline	Loading Condition	Limitations	Limit State	Model
ACI 440.2R-17	AC AC + B	SR-section: *h*/*b* ≤ 2.0; *h* or *b* ≤ 900 mm; fully wrapped only	ULS SLS	Lam and Teng [15]
S806-12	AC + B	SR-section: *h*/*b* ≤ 1.5; *R* ≥ 20 mm; fully wrapped only	ULS	not specified
CNR-DT 200 R1/2013	AC AC + B	SR-section: *h*/*b* ≤ 2.0; *h* or *b* ≤ 900 mm; discontinuous wrapping: *s* ≤ *D*/2	ULS	not specified
GB 50608-2010	AC + B	SR-section: *h*/*b* ≤ 1.5; *h* or *b* ≤ 600 mm; *R* ≥ 20 mm; fully wrapped only	ULS	Teng et al. [25]; Jiang [17]
DAfStb-Guideline	AC + B	circular only: *D* ≥ 120 mm; *λ* ≤ 40; *e*_0_/*D* ≤ 0.25 *f*_c_ ≤ 58 N/mm²; fully wrapped only	ULS SLS	Niedermeier [26]; Jiang [17]

Abbreviations: AC = pure axial compression; AC + B = combined axial compression and bending; ULS = ultimate limit state; SLS = serviceability limit state; SR = noncircular cross section; *R* = corner radius; *h* = height and *b* = width for rectangular cross sections, s = net distance between FRP strips, *D* = diameter of circular cross section, *λ* = column slenderness, *e*_0_/*D* = maximum eccentricity, *f*_c_ = unconfined concrete strength.

**Table 3:** Strength reduction and material safety factors.

Standard/Guideline	Input Value	Strength Reduction or Partial Safety Factor	Partial Safety Factor Environment	Additional Factor
ACI 440.2R-17	mean values	*φ* = 0.75 (spiral); *φ* = 0.70 (steel-tie)	*C*_E_ for C, G, or A under I, E, or Agg	*Ψ*_f_ = 0.95
S806-12	mean values	*γ*_j_ = 0.75; *γ*_c_ = 0.65; *γ*_s_ = 0.85	not used	not used
CNR-DT 200 R1/2013	characteristic values	*γ*_j_ = 1.10; *γ*_c_ = 1.50; *γ*_s_ = 1.15	*η*_a_ for C, G, or A under I, E, or Agg	*γ*_Rd_ = 1.10
GB 50608-2010	characteristic values	*γ*_j_ = 1.40	*γ*_e_ for C, G, B, or A under I, E, or Agg	not used
DAfStb-Guideline	characteristic values	*γ*_j_ = 1.35; *γ*_c_ = 1.50; *γ*_s_ = 1.15	*α*_T_ = 0.70 (temperature); *α*_E_ = 1.00 (loading); *α*_F_ = 1.00 (moisture); *α*_Z_ = 0.75 (loading duration)	*α*_cc_ = 0.85

Abbreviations: C = Carbon, G = Glass, A = Aramid, B = Basalt, I = interior, E = exterior, Agg = aggressive environment. *γ*_j_ = safety factor FRP jacket, *γ*_c_ = safety factor concrete, *γ*_s_ = safety factor steel, *γ*_Rd_ = safety factor to consider model uncertainties, *α*_cc_ = further conversion factor for concrete, *C*_E_, *η*_a_, and *γ*_e_ = environmental reduction factors according to particular code, *α*_T_, *α*_E_, *α*_F_, and *α*_Z_ = reduction factors to address environmental as well as loading conditions according to DAfStb-Guideline, *Ψ*_f_ = additional reduction factor.

**Table 4:** Efficiency factor *k*_ε_ and complementary limits for predictions of the hoop strain *ε*_ju_ in FRP jacket.

Standard/Guideline	Efficiency Factor *k*_ε_	Characteristic Value *k*_εk_	Rupture Hoop Strain Model	Further Limit
ACI 440.2R-17	0.55	not used	*ε*_ju_ = *k*_ε_ · *ε*_FRP_ · *C*_E_	*ε*_ju_ ≤ 0.004 if AC + B
S806-12	1.00	not used	*ε*_jud_ = *γ*_j_ · *ε*_FRP_	*ε*_jud_ ≤ 0.006
CNR-DT 200 R1/2013	1.00	not used	*ε*_jud_*= η*_a_ · *ε*_FRP,k_/*γ*_j_	*ε*_jud_ ≤ 0.004
GB 50608-2010	0.50 (CFRP) 0.70 (GFRP)	not used	*ε*_jud_*= k*_ε_ · *ε*_FRP,k_/(*γ*_j_ · *γ*_e_)	not used
DAfStb-Guideline	0.50	0.25	*ε*_juk_*= k*_εk_ · *ε*_FRP,k_ · *α*_T_ · *α*_E_ · *α*_F_ · *α*_Z_	not used

Abbreviations: CFRP = carbon fiber reinforced polymer, GFRP = glass fiber reinforced polymer, AC + B = combined axial compression and bending, *ε*_FRP_ = mean value of maximum strain for an FRP sheet, *ε*_FRP,k_ = characteristic maximum strain for an FRP sheet, *ε*_juk_ = characteristic hoop rupture strain in FRP jacket at column, *ε*_jud_ = design hoop rupture strain.

**Table 5:** The factor *k*_1_ determined in the considered codes and guidelines.

Standard/Guideline	Factor *k*_1_	Factor *k*_1k_	Particularities
ACI 440.2R-17	3.30	not used	additional, *k*_1_ is multiplied with *Ψ*_f_
S806-12	6.70 · (*f*_lj_)^−0.17^	not used	no
CNR-DT 200 R1/2013	2.60	not used	instead of *f*_lj_, *k*_1_ is multiplied with *f*_cd_ · (*f*_ljd_/*f*_cd_)^2/3^
GB 50608-2010	3.50	not used	additional, *k*_1_ is multiplied with (1–6.5/*β*_j_); *β*_j_ = (*E*_j_ · *t*_j_)/(*f*_ck_ · *D/2*)
DAfStb-Guideline	3.66	2.00	instead of *f*_lj_, *k*_1_ is multiplied with (*f*_ljk_ + (*ρ*_w_ · *f*_yk−_Δ*p*) · *k*_e_); *k*_e_ = ((*D*_c_−*s*/2)/*D*)^2^

Abbreviations: *f*_ck_ = characteristic concrete strength, *f*_cd_ = design concrete strength, *f*_ljk_ = char. FRP jacket strength, *f*_ljd_ = design FRP jacket strength, *ρ*_w_ = ratio of transverse reinforcement, *f*_yk_ = is the characteristic value of yield strength of transverse steel, Δ*p* = pressure gradient between internal reinforcement and FRP jacket, *D*_c_ = diameter of core of section enclosed by transverse reinforcement, *s* = center to center spacing of circular hoop.

**Table 6:** Approaches suggested in codes and guidelines to predict the maximum concrete strain.

Standard/Guideline	Approach	Factors/Particularities
ACI 440.2R-17	Equation (2)	*k*_2_ = 1.50, *k*_3_ = 12, *k*_4_ = 0.45; *ε*_ccu_ ≤ 0.01
S806-12	not provided (refer to CAN/CSA-A23.3→*ε*_ccu_ = 0.0035)	no
CNR-DT 200 R1/2013	$\varepsilon_{\mathrm{ccu}}\;=\;0.0035\;+\;0.015\;\cdot \;\sqrt{\frac{f_{\mathrm{ljd}}}{f_{\mathrm{cd}}}}$	for calculation of *f*_ljd_→$\varepsilon_{\mathrm{jud}}\;\leq \;0.6\;\cdot {\;\varepsilon }_{\mathrm{FRP},k}$
GB 50608-2010	$\varepsilon_{\mathrm{ccu}}\;=\;0.0033\;+\;0.60\;\cdot {\;\beta }_{j}^{0.8}\;\cdot {\;\varepsilon }_{\mathrm{jud}}^{1.45}$	no
DAfStb-Guideline	Equation (2)	*k*_2_ = 1.75, *k*_3_ = 19, *k*_4_ = 0; in Equation (2): (*f*_lj_/*f*_c_) is replaced by (*f*_ljk_/*f*_c_)

Abbreviations: *f*_ck_ = characteristic concrete strength, *f*_cd_ = design concrete strength, *f*_ljk_ = characteristic FRP jacket strength, *f*_ljd_ = design FRP jacket strength, $\epsilon_{\mathrm{FRP},\;k}$ = characteristic maximum strain for an FRP sheet.

## References

[1] Matthys S., Toutanji H., Audenaert K., Taerwe L. (2005). Axial Load Behavior of Large-Scale Columns Confined with Fiber-Reinforced Polymer Composites. ACI Struct. J..

[2] Lee J.-Y., Oh Y.-J., Park J.-S., Mansour M.Y. Behavior of Concrete Columns Confined with Steel Spirals and FRP Composites. Proceedings of the 13th World Conference on Earthquake Engineering.

[3] Lam L., Teng J.G. (2004). Ultimate Condition of Fiber Reinforced Polymer-Confined Concrete. J. Compos. Constr..

[4] Xiao Y., Wu H. (2003). Compressive Behavior of Concrete Confined by Various Types of FRP Composite Jackets. J. Reinf. Plast. Compos..

[5] Eid R., Roy N., Paultre P. (2009). Normal- and High-Strength Concrete Circular Elements Wrapped with FRP Composites. J. Compos. Constr..

[6] Rousakis T.C., Rakitizis T.D., Karabinis A.I. (2012). Design-Oriented Strength Model for FRP-Confined Concrete Members. J. Compos. Constr..

[7] Rousakis T.C., Karabinis A.I. (2012). Adequately FRP confined reinforced concrete columns under axial compressive monotonic or cyclic loading. Mater. Struct..

[8] Achillopoulou D.V., Rousakis T.C., Karabinis A.I. Square Reinforced Concrete Columns Strengthened Through Fiber Reinforced Polymer (FRP) Sheet Straps. Proceedings of the 6th International Conference on FRP Composites in Civil Engineering (CICE 2012).

[9] Lin G., Teng J.G. (2019). Stress-Strain Model for FRP-Confined Concrete in Eccentrically Loaded Circular Columns. J. Compos. Constr..

[10] Zeng J.J., Lin G., Teng J.G., Li L.J. (2018). Behavior of large-scale FRP-confined rectangular RC columns under axial compression. Eng. Struct..

[11] Ferrotto M.F., Fischer O., Niedermeier R. (2017). Experimental investigation on the compressive behavior of short-term preloaded carbon fiber reinforced polymer-confined concrete columns. Struct. Concr..

[12] Pan Y., Rui G., Li H., Tang H., Xu L. (2017). Study on stress-strain relation of concrete confined by CFRP under preload. Eng. Struct..

[13] Jiang C., Yuan F., Wu Y.-F., Zhao X.-M. (2019). Effect of Interfacial Bond on Plastic Hinge Length of FRP-Confined RC Columns. J. Compos. Constr..

[14] Wu Y.-F., Jiang C. (2013). Effect of load eccentricity on the stress-strain relationship of FRP-confined concrete columns. Compos. Struct..

[15] Lam L., Teng J.G. (2003). Design-Oriented Stress-Strain Model for FRP-Confined Concrete in Rectangular Columns. J. Reinf. Plast. Compos..

[16] Jiang T., Teng J.G. (2013). Behavior and Design of Slender FRP-Confined Circular RC Columns. J. Compos. Constr..

[17] Jiang T. (2008). FRP-Confined RC Columns. Analysis, Behavior and Design. Ph.D. Thesis.

[18] (2012). M/515 EN. Mandate for Amending Existing Eurocodes and Extending the Scope of Structural Eurocodes. https://eurocodes.jrc.ec.europa.eu/doc/mandate/m515_EN_Eurocodes.pdf.

[19] (2004). Eurocode 2: Design of Concrete Structures.

[20] (2012). Design and Construction of Building Structures with Fibre-Reinforced Polymers.

[21] (2013). CNR-DT 200 R1/2013. Guide for the Design and Construction of Externally Bonded FRP Systems for Strengthening Existing Structures. https://www.cnr.it/en/node/2636.

[22] (2010). Technical code for Infrastructure Application of FRP Composites.

[23] (2012). DAfStb-Richtlinie Verstärken von Betonbauteilen mit geklebter Bewehrung.

[24] (2017). Guide for the Design and Construction of Externally Bonded FRP Systems for Strengthening Concrete Structures.

[25] Teng J.G., Jiang T., Lam L., Luo Y.Z. (2009). Refinement of a Design-Oriented Stress-Strain Model for FRP-Confined Concrete. J. Compos. Constr..

[26] Niedermeier R. (2009). Verstärkung von Stahlbetondruckgliedern durch Umschnürung. Ph.D. Thesis.

[27] (2016). FIB Bulletin No. 80. Partial Factor Methods for Existing Concrete Structures.

[28] Smith S.T., Kim S.J., Zhang H. (2010). Behavior and Effectiveness of FRP Wrap in the Confinement of Large Concrete Cylinders. J. Compos. Constr..

[29] Toutanji H., Matthys S., Taerwe L., Audenaert K. Behaviour of large-scale columns confined with FRP composites in compression. Proceedings of the 2nd International Conference on FRP Composites in Civil Engineering (CICE 2004).

[30] Pellegrino C., Modena C. (2010). Analytical Model for FRP Confinement of Concrete Columns with and without Internal Steel Reinforcement. J. Compos. Constr..

[31] Samaan M., Mirmiran A., Shahawy M. (1998). Model of Concrete Confined by Fiber Composites. J. Struct. Eng..

[32] Cui C., Sheikh S.A. (2010). Analytical Model for Circular Normal- and High-Strength Concrete Columns Confined with FRP. J. Compos. Constr..

[33] Hu H., Seracino R. (2014). Analytical Model for FRP-and-Steel-Confined Circular Concrete Columns in Compression. J. Compos. Constr..

[34] Eid R., Paultre P. (2008). Analytical Model for FRP-Confined Circular Reinforced Concrete Columns. J. Compos. Constr..

[35] Teng J.G., Lin G., Yu T. (2015). Analysis-Oriented Stress-Strain Model for Concrete under Combined FRP-Steel Confinement. J. Compos. Constr..

[36] (2012). Deutscher Ausschuss für Stahlbeton e. V. Erläuterungen zu DIN EN 1992-1-1 und DIN EN 1992-1-1/NA (Eurocode 2).

[37] Käseberg S. (2016). Verstärkung von Stahlbetonstützen mit Kreisquerschnitt durch Umschnürung mit CFK-Werkstoffen. Ph.D. Thesis.

[38] Käseberg S., Holschemacher K. (2015). Dual Confinement of Circular Concrete Columns Consisting of CFRP Sheets and Steel Ties or Spirals. J. Civil. Eng. Archit. Res..

[39] Käseberg S., Holschemacher K., Curbach M. (2018). Zum Tragverhalten CFK-umschnürter Stahlbetonstützen mit Kreisquerschnitt. Beton- und Stahlbetonbau.

[40] Bai Y.-L., Dai J.-G., Teng J.G. (2017). Buckling of steel reinforcing bars in FRP-confined RC columns: An experimental study. Constr. Build. Mater..

